# Medical Opinion and Movement

**Published:** 1908-03-14

**Authors:** 


### Medical Opinion and Movement.

INFLUENZA has been so prevalent during the last few weeks in most parts of the country as to constitute a veritable epidemic, reminding one of those of some years back. To a certain extent it is an annual visitation, but the disease has shown during the last few years signs of diminution both in extent and virulence. It has been suggested that the community is gradually undergoing a process of immunisation. This may be so, but the disease is one which does not appear to confer much immunity upon its victims even by many attacks. It is, however, generally admitted that, although the disease is widespread, it has not assumed a very virulent form, but has for the most part conformed to the milder type, such as one is accustomed to meet at all times. The acute symptoms usually subside in two or three days, and the patient is frequently satisfied to treat this part of the disease by rest in bed and a bottle of ammoniated tincture of quinine. He is surprised to find, however, that although the febrile symptoms have left, he is not well. Wearied with a bronchial, troublesome cough, feeble in body, and depressed in spirits, he sends for his doctor. Even after such mild attacks the general depression is often very marked. Abdominal cases appear to be less prevalent than in previous outbreaks.

"TYPHOID bacillus carriers" is a subject which occupies at the moment a foremost place of interest in current medical literature. Instances of outbreaks of fever, especially in asylums, traced to the existence of one or more typhoid carriers in the midst of the community, are multiplying, and in future epidemics this source of infection will always doubtless be borne in mind. The question has already been fully discussed in these columns, and it was pointed out that opinion was in favour of the gall-bladder as the seat of infection, where the microbes continue to live and flourish and thence make their way into the intestine and pass out with the fæces. It has been ascertained that the bacillus typhosus not only shows a selective power for the gall-bladder but also tends to persist there after the attack of the fever. Gall-bladder troubles not infrequently occur after the disease as well as during the attack itself, and a connection has been made out by some authorities between typhoid infection of the gall-bladder and the formation of gall-stones. An interesting case just reported by Dr. George Dean, of the Lister Institute, offers further support to this theory. The patient, a doctor, had a severe attack of typhoid fever twenty-nine years ago. Since then he has been subject to one or more attacks of biliary colic every year, sometimes accompanied by jaundice. It occurred to Dr. Dean that the case might be one of cholecystitis typhosa, and a thorough bacteriological examination of the stools was made, with the result that the typhoid bacilli were found in abundance, to the exclusion to a large extent of other organisms usually present in the fæces.

DURING the last two years, owing largely to the work of Förster and his pupils at Strassburg, our conception of the pathogenesis of typhoid fever has had to undergo considerable modification, and the chain of events supposed to be involved in the invasion of the human subject by the typhoid bacillus must be to a large extent reversed. Formerly it has been pictured that the typhoid bacillus first finds its way into the alimentary canal, multiplies there, and then invades the tissues, chiefly the lymphoid tissues of the small intestine and the spleen. The invasion of the blood stream, setting up a septicæmic condition, was supposed only to take place as a secondary event, generally at a later stage of the disease. But Conradi has shown that the bacilli are present in the blood during the incubation period, and Fornet has found by the precipitin test the presence of anti-bodies even before the bacilli in the blood. On the other hand, bacilli do not appear in the fæces until the first and second weeks of the disease. Förster therefore pictures the course of the invasion briefly as follows: The bacilli enter the body by the digestive tract, possibly by the throat, setting up in some cases a typhoid angina; thence they pass rapidly into the blood, and so arrive at the gall-bladder, whence they are eventually expelled into the intestine. In most cases recovery is accompanied by a cessation of the inflammatory processes in the liver, bile-ducts, and gall-bladder, but in about 2 per cent. of the cases a cholecystitis typhosa persists, giving rise to subsequent troubles and constituting the chronic typhoid carrier with typhoid infected stools.

THE causal relations between accident and the development of a hernia is a question of considerable importance in regard to workmen's compensation. Rupture has always been associated in the popular mind with some accident of force or strain. Among medical authorities opinion has for some time been growing in favour of the belief that in a large proportion of cases hernia is the result of a slow process, the true origin of which is a constitutional defect in the abdominal wall. One group of writers, Kingdon among them, has gone so far as to hold that hernia is always a disease, never an accident. One can only admit the possible truth of this statement in so far that we may probably assume in almost all cases the existence of a preformed congenital sac. It must be recognised that this sac might be carried empty to the grave in many cases were it not for the coexistence of some exciting cause in the way of a strain or unusual effort. Dr. William B. Coley, of New York, has recently reviewed this question, and after consideration of statistics and the opinions of others he agrees with Berger and Murray in the view that in practically all cases of hernia, with exception of direct inguinal and some femoral herniæ, there is present from birth a preformed sac or peritoneal pouch as the great predisposing cause. A predisposition is, however, not sufficient to exclude compensation, although it may materially reduce it if it can be shown that the exciting cause was insignificant. Such relative conditions can only be adjudged in the individual cases. From a medical point of view special consideration must be given to the circumstances connected with the accident, the personal antecedents of the individual, the symptoms immediately after the accident, the nature of the hernia itself, and the condition of the other hernial orifices and of the abdominal wall in general.

Rectal neuralgia is a condition which is treated of very briefly even in large text-books, though various American authors have from time to time drawn attention to its occurrence in alcoholic and neurasthenic patients. Albu, who has made a study of five cases which came under his personal observation, doubts if the affection is of rheumatic origin as some observers have maintained. Usually the only objective symptom is a heavily laden lower bowel, and the author is of opinion that chronic constipation is probably the main cause in most cases. Subjectively the patients complain of severe and sometimes paroxysmal pains which radiate from the anus to neighbouring parts. Men are much more commonly affected than women, and the pains are definitely neuralgic, accompanied with pain on pressure over the course of the perineal nerves. The diagnosis must be made solely by careful elimination of other causes of rectal pain, chief among which are coccyodynia, rectal crises in locomotor ataxia, the pains of rectal cancer, and the neuritises caused by pressure of tumours, etc., in this region. The treatment is directed towards improving the general health, maintaining a free action of the bowels, and combating the excruciating pains when they occur. The author has not found narcotics of much use for this last purpose, and prefers belladonna, either by mouth or by rectal injections. Forcible stretching of the perineal nerves by manipulation and the introduction of bougies sometimes succeeds when all other methods of cure appear useless.

There have hitherto been comparatively few published observations on that rare accident, volvulus of the stomach. An interesting case is reported by Niosi in an Italian contemporary. The patient, a woman of thirty-eight, who had suffered since childhood from dyspepsia, was admitted to the surgical clinic at Pisa for violent gastric pain. The acute symptoms, intense epigastric pain and tenderness, persistent vomiting, nausea, and collapse had come on three days before admission. On examination a hard ovoid swelling, the size of a hen's-egg, was found, apparently at the pyloric end of the stomach. The stomach itself was dilated. At the operation an annular constriction of the stomach was found, together with signs of old inflammation of the omentum. The lump which had been felt before operation was due to a cicatrisation of an old ulcer at the pyloric end round which the omentum had become firmly adherent. In addition the pyloric portion of the stomach was found to have undergone a degree of torsion, with the result that the natural position was reversed, the anterior wall looking directly backwards and the great curvature being superior, while the lesser curvature looked downwards. The volvulus was rectified, and as the communication between the two divisions of the stomach was fairly free, a posterior gastroenterostomy was done. The patient made an excellent recovery. In discussing the case, Niosi differentiates between total volvulus, in which the whole organ undergoes axial rotation, and partial volvulus, in which the pyloric end only is twisted. The reported cases which belong to the second category are few, and among them must be placed M. Niosi's recent case. The ætiology of the condition is obscure. In his case, the author thinks that the degree of dilatation of the stomach, together with the pyloric stenosis which was found to exist at the operation, suffices to explain the causation.

Lewandowski, in the *Zeitschrift f.aerztliche Fortbildung*, summarises the indications for "respiratory gymnastics" or systematic and regulated breathing exercises as an aid in the treatment of various complaints. The exercises must be regulated by the physician, and the author recommends a variety of movements, chief among which he regards the regular forcible expiration and inspiration in the open air, the patient standing with the head thrown back and the arms extended. Such exercises are specially suited for patients predisposed to pulmonary tubercle. In those in which an apical lesion has been definitely diagnosed, great care must be taken in regulating the exercises, on account of the danger of causing strain and bringing on an attack of hæmoptysis. In emphysematous conditions special stress is laid on the importance of expiratory breathing, and the author warns against recommending the treatment in cases of pleurisy before all inflammatory reaction has disappeared. In combination with other active and passive movements the author has found respiratory gymnastics of great value in the treatment of chronic heart cases, in diseases of metabolism, such as diabetes, gout, and obesity, and in digestive troubles.

In the course of an extremely interesting article in a recent number of *La Semaine Medicale*, Professor de Bovis, of Rheims, discusses the post-operative treatment of laparotomy cases. It is nearly four years ago since Boldt, in America, tried to answer the question, "For how long a time should we enjoin rest in bed in cases of abdominal section?" by laying down a general rule that all such cases should be allowed to get up after the first week. Other surgeons have not followed this revolutionary treatment, though it is indubitable that there is a general tendency, more apparent in America than in France, to abbreviate as much as possible the lying in bed after laparotomies. Dr. Riess of Chicago was, we believe, the first to draw the attention of the profession generally to the undesirabilty of enjoying a lengthened stay in bed after severe operations, but in 1899 surgeons were not prepared to admit his premises, and the treatment he proposed never became popular. Even before Riess, Robert Morris had allowed his patients to get up as soon as they felt strong enough to do so —usually within fifteen days of the operation—and the results he gained were decidedly encouraging.

PROFESSOR DE BOVIS, in his able paper, discusses the whole question in an impartial and elucidative manner. He adopts what is virtually the Riess method of treatment. After a laparotomy the patient is bandaged in a special manner, the bandage being carried high up and made to envelop the whole of the abdomen, forming, in fact, a support to the abdominal walls. So bandaged the patient is allowed to get up when he likes. "Usually," remarks Professor de Bovis, "he does not desire to avail himself of the permission before the fourth day, when the immediate effects of the operation and the anæsthetic have passed off," but, like Boldt, he would not hesitate to allow the patient to get up twenty-four hours afterwards if the latter had any desire to do so. Only rest on a couch is allowed during the first few days, but after a week the patient may get on his feet and may walk, due care being taken to prevent him from over-exerting himself, and after a fortnight's stay in hospital the patient is usually quite fit to go home.

THE advantages of this post-operative abulatory treatment are obvious. It greatly shortens the period of convalescence, the patient runs far less risk of hypostatic pneumonia, intestinal and bladder inconveniences, by no means unusual when the ordinary method of post-operative treatment is followed are avoided, and finally it greatly improves the general nutrition of the patient. This last advantage is specially marked in cases of hysterectomy, done for fibroids which, through metrorrhagia, have greatly weakened the patient and rendered her profoundly anæmic. In such cases the ambulatory treatment brings about a striking amelioration in the general condition of the patients. The disadvantages and contra-indications of the new method, however, should not be lost sight of. Septic cases, those in which delicate plastic operations have been done on the abdominal viscera, and operations on very feeble and ill-nourished patients cannot be treated in this manner. In many cases, too, chloroform narcosis leaves the patients in a very precarious state, but this contra-indication, as Professor Kroenig thought, may largely be obviated by the use of local anæsthesia. The ambulatory treatment is therefore only suitable for selected cases, and even in these there are two great risks which the surgeon should take into consideration. The first is the risk of embolism and post-operative phlebitis, a risk which the series of statistics adduced by Professor de Bovis shows to be by no means a small one. Secondly, there is the danger that the sutures may give way, leading to distressing ventral herniæ and, in careless hands, to the greater risk of septic infection of the wound. On the face of it the theory that primary union is accelerated by constant movement of the parts seems absurd, but it is possible that this second objection has weighed too much with surgeons in their treatment of such cases. The whole question of the post-operative treatment in cases of abdominal section is one that should be considered from the point of view of each individual case, and the ambulatory method of treatment should certainly be considered, its objections and i[illegible] advantages being carefully weighed before it is give[illegible] a trial, in each case that appears to be suitable.

CALMETTE and Breton, in a communication t[illegible] the Biological Society of Paris, state that the[illegible] have been experimenting with rectal injections c[illegible] tuberculin. Their results have been interesting especially in view of the recent observations re[illegible]garding the absorption by the rectal mucos[illegible] of snake venom, cholesterin and other substances by certain French investigators. The tuberculi[illegible] was given in the form of milk enemata, each con[illegible]taining .01 centigram of tuberculin. In four case[illegible] the injections were followed by a characteristi[illegible] febrile reaction, and the reappearance of th[illegible] ophthalmo-tuberculin reaction. Similar injections given to non-tubercular patients, were absolutel[illegible] negative, no ophthalmo-reaction being obtainable.

AN interesting discussion was evoked at a recen[illegible] meeting of the Paris Surgical Society as to th[illegible] functional activity of the pylorus after gastro jejunostomy. M. Legueu showed a case in whicl[illegible] he had performed a gastro-enterostomy, two year[illegible] ago, for gastric trouble. At the operation no sig[illegible] of pyloric contraction or stenosis was found. The result of the operation was that the patient was relieved of all his symptoms, and it was found, on radioscopic examination, that "the anastomotic opening was functioning perfectly." Professor Hartmann, in discussing the case, doubted the possibility of finding out by means of the $x$-rays, whether or not the pylorus was functioning at the same time, and stated that he saw no reason to change his opinion that a gastro-jejunostomy was useless except in cases where there was definite pyloric obstruction. Professor Montprofit and other speakers, however, supported M. Legueu, stating that they had had excellent results from the operation in cases where it was impossible to doubt the patency of the pylorus. M. Montprofit declared that in two of his cases he had satisfied himself that the anastomotic opening was functioning well, though there was no stenosis of the pyloric end of the stomach.

JANOWSKI of Warsaw has lately made an exhaustive study of the value of cytological examination of pleural exudates, and his conclusions tend to demonstrate that such examinations may be of great value in the diagnosis of obscure conditions. In twenty-eight cases, in which the effusion was undoubtedly tuberculous, he found a decided increase in the amount of lymphocytes present, thus confirming the observations made eight years ago by Widal and Ravaut. But Janowski is disinclined to believe that his observations allow him to diagnose definitely, in cases where an increase of lymphocytes is found, that the effusion is necessarily tuberculous. Such evidence is strongly presumptive of a tuberculous lesion, and where other considerations support the diagnosis, it may be of value in eliminating other causes. The subject, however, is one which stands in need of further elucidation.

